# DomHR: Accurately Identifying Domain Boundaries in Proteins Using a Hinge Region Strategy

**DOI:** 10.1371/journal.pone.0060559

**Published:** 2013-04-11

**Authors:** Xiao-yan Zhang, Long-jian Lu, Qi Song, Qian-qian Yang, Da-peng Li, Jiang-ming Sun, Tong-hua Li, Pei-sheng Cong

**Affiliations:** Department of Chemistry, Tongji University, Shanghai, China; Indian Institute of Science, India

## Abstract

**Motivation:**

The precise prediction of protein domains, which are the structural, functional and evolutionary units of proteins, has been a research focus in recent years. Although many methods have been presented for predicting protein domains and boundaries, the accuracy of predictions could be improved.

**Results:**

In this study we present a novel approach, DomHR, which is an accurate predictor of protein domain boundaries based on a creative hinge region strategy. A hinge region was defined as a segment of amino acids that covers part of a domain region and a boundary region. We developed a strategy to construct profiles of domain-hinge-boundary (DHB) features generated by sequence-domain/hinge/boundary alignment against a database of known domain structures. The DHB features had three elements: normalized domain, hinge, and boundary probabilities. The DHB features were used as input to identify domain boundaries in a sequence. DomHR used a nonredundant dataset as the training set, the DHB and predicted shape string as features, and a conditional random field as the classification algorithm. In predicted hinge regions, a residue was determined to be a domain or a boundary according to a decision threshold. After decision thresholds were optimized, DomHR was evaluated by cross-validation, large-scale prediction, independent test and CASP (Critical Assessment of Techniques for Protein Structure Prediction) tests. All results confirmed that DomHR outperformed other well-established, publicly available domain boundary predictors for prediction accuracy.

**Availability:**

The DomHR is available at http://cal.tongji.edu.cn/domain/.

## Introduction

Protein domains are the structural, functional and evolutionary units of proteins. A domain is a segment of a polypeptide chain that can fold into a compact and stable three-dimensional structure independently of other segments in the chain [1]. Most domains are single continuous polypeptide segments, while a few consist of several discontinuous segments. Small proteins often consist of only a single domain, while many large proteins comprise two or multiple structural domains [2]. Domains can function independently or they can work with neighboring domains in a harmonious way. Domains are the building blocks of proteins in molecular evolution, allowing different arrangements and reorganizations to create proteins of different functions. Therefore, the exact identification of protein domains and their boundaries is important not only for protein classification and the study of protein structure, function, and evolution, but also for drug discovery, disease treatments and genetic engineering. Unfortunately, experimental methods for the identification of protein domain boundaries are time consuming and labor intensive. In addition, large numbers of protein sequences are being generated. The speed of manual identification and annotation of proteins lags behind the rate of sequence creation. To fill this gap, a computational approach to domain identification is highly desirable.

Identifying domains and boundaries requires a clear definition of each; however, domain and boundaries are ambiguously defined in the literatures [3], [4], [5], [6], [7]. Three main criteria were used to decide if a protein structure should be parsed into smaller domains and to determine the domain boundaries: geometrical separation, symmetry, and recurrence in other structures [8]. However, domains assigned manually by visual inspection and automatic domain-parsing programs were quite different [9]. Even in SCOP and CATH, the two most commonly used databases of protein structures, the definitions of domains vary, as does hierarchic partitioning of the fold space on each level [10]. The different definitions of domain from structural, functional and evolutionary information have been used to develop many methods of domain predictions.

Currently, studies about protein domain boundary prediction are divided into two categories: those that use template-based methods [11], [12], [13], [14], [15], [16] and those that use ab-initio methods [17], [18], [19], [20], [21], [22]. Template-based methods generally use alignment against a known domain database (such as CATH [11] or SCOP [23]) to predict domain boundaries. For example, the sequence or secondary structures of a target sequence might be aligned against the sequence or the secondary structures in a domain classification database [24], [25]. The performance of template-based method is more accurate than other methods. However, this performance is dependent on the homology between the target and the known structures. The ab-initio method has no such restrictions. Ab-initio methods generate a learning model using machine learning technologies that use a series of proteins for which information on residue properties is known. In this strategy, artificial neural networks (ANN) [26] and support vector machines (SVM) [27] are the most widely used algorithms.

Among these algorithms, Liu and Rost [28] used ANN with evolutionary information, amino acid composition, predicted secondary structure, predicted solvent accessibility, and amino acid flexibility. The networks used by Nagarajan and Yona [29] were merged with the information of multiple sequence alignments analysis and position-specific physiochemical properties of amino acids and predicted secondary structures. PPRODO [30] used information from a position-specific scoring matrix (PSSM) generated by PSI-BLAST [31] in an ANN. DOMpro [3] applied a 1D recursive neural network to incorporate evolutionary information in the form of profiles, and predicted secondary structure and solvent accessibility. Ye et al. [32] used a back-propagation ANN method with various sequence profiles, based on chemical, physical, and statistical properties. Yoo et al. [33] used an improved general regression network model trained by the information of a PSSM, an interdomain linker index, secondary structure, and solvent accessibility.

In recent years, SVM has been used by DomainDiscovery [34] to predict domain boundaries with sequence information including a PSSM, a secondary structure, solvent accessibility information and an interdomain linker index. DomSVR [35] predicted domain boundaries using SVR starting from the protein sequence alone and using only profiles generated from an AAindex database [36]. DROP [4] developed an SVM to predict domain linkers using 25 optimal features selected from a set of 3000 features including PSSMs and over 2000 physicochemical properties via a random forest algorithm. DoBo [5] used the classification capability of SVM to improve protein domain boundary prediction using evolutionary domain boundary signals embedded in homologous proteins. Cai et al. [37] employed a random forest algorithm, maximum relevance minimum redundancy, and incremental feature selection, incorporating the sequence conservation, residual disorder, secondary structure propensity, and solvent accessibility to predict domains.

Here, we present a novel approach, DomHR, which is an accurate predictor of protein domain boundaries based on a creative hinge region strategy. DomHR uses a nonredundant dataset as the training set, domain-hinge-boundary (DHB) and predicted shape strings as features, and a conditional random field as the classification algorithm. In predicted hinge regions, a residue is determined as domain or boundary according to the decision threshold. DomHR is a hybrid method such as DOMAC [38] or method proposed by Walsh [39]. After the decision thresholds were optimized, DomHR was tested in cross validation, large-scale prediction, independent test and CASP tests.

## Materials and Methods

### Datasets

Three datasets (S628, S3845 and S1508) and one database (B25936) were used in this study. S628 was extracted from Cheng’s package [5], in which the sequence identity of each pair of protein chains was less than 25%, the domain number of the proteins agreed in both SCOP (v 1.75) and CATH (v3.3.0), and any protein whose length was less than 90 residues was removed. This resulted in a final dataset containing 628 protein sequences, of which 186 were multidomain proteins and 442 were single-domain proteins. The domain definitions were those provided by CATH.

The key database with sequence and domain/boundary information was derived from the annotated domains in the CATH and PDB (Protein Data Bank) [40]. First, 138,550 IDs of protein sequences were collected from CATH version 3.5.0, and the corresponding sequences were extracted from PDB. These sequences were sorted according to the number of domains and the lengths of boundaries in descending order. Sequences with more domains and longer boundaries were easy retained after redundant sequences were removed. Next, PISCES [41] was carried out to reduce sequence redundancy in the data by ensuring that sequence identity was limited at 99% and short sequences (<40 residues) were filtered out. The remaining database (B25936) contained 25,936 entries in which sequences and DHB information were joined. Data redundancy was further reduced using PISCES at 25% sequence identity, resulting in 5353 entries. Among these, 3845 entries that appeared in both CATH 3.4.0 and CATH 3.5.0 were collected as the training set (S3845). The remaining entries (1508) were collected as the independent testing set (S1508), in which each sequence was assigned a domain definition by CATH 3.5.0.

### Domain Boundary Definitions

Similar with the different definitions of domain, as mentioned above, domain boundary definitions also vary. Zou [7] defines a domain boundary as the residues between SCOP domains. DROP [4] defines a domain linker as a loop region separating two structural domains without α-helices or β-strands. DOMpro [3] defines residues within 20 amino acids of a domain boundary as domain boundary residues, with all other residues considered as nonboundary residues. David [6] broadened the boundary definition to a neighborhood of 10 amino acids. Cheng [5] recognized the first and the end residues of a domain and the linker as domain boundaries.

**Figure 1:**
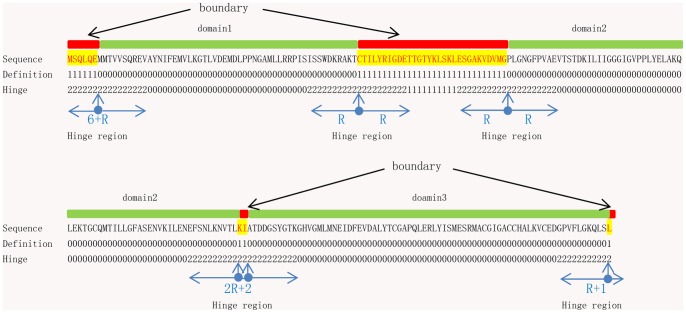
Definitions of domain boundaries and hinge regions. The red represents boundary, the green represents domain. A residue assigned to the boundary is coded with “1″; A residue assigned to the hinge region is coded with “2″; otherwise, “0″. A hinge region contains 2*R (for example, R = 10) residues that are centrally located at the N- and C-terminus of a domain and bidirectionally extended into the domain and the boundary R residues.

In this study, the domain boundary was defined as the nondomain residue defined by CATH and the N- and C-terminal residues of a domain (Figure 1). In addition, when two domains were connected to each other, we defined two consecutive contacted residues as boundary elements. Thus the start and end residues of a domain always were defined as boundaries. This definition slightly increased the numbers of boundaries. However, this was expected to be useful for domain identification, especially when two domains were connected.

### Definition of Hinge Region

Recently, binary classification has tended to be framed as a three-class problem. Cheng [5] defined false boundaries, near boundaries, and away boundaries, and constructed two predictors to identify domain boundaries. Zhou [42] defined ordered residues, and short and long disordered regions in his predictor, and reduced the three-state model to a two-state prediction.

In this study, we defined a hinge region as containing 2*R (for example, R = 10) residues that are centrally located at the N- and C-terminus of a domain and bidirectionally extended into the domain and the boundary R residues (Figure 1). An additional hinge region could improve the imbalance between the positive and negative data without removing any residues. In a hinge region, where predicted errors often occur, we analyzed and adjusted decision thresholds to improve the performance of the predictor (see below).

### Profiles of Domain, Hinge and Boundary

The novel technology in this study was DHB profiles. The DHB feature was generated by sequence-domain/hinge/boundary alignment. For a query, sequence alignment was carried out first against B25936 using PSI-BLAST [31] (the parameters were set as: Number of iterations = 2; other parameters set as default) to find sequences homologous to the target sequence. Matched piecewise local sequences were then selected according to *e*-values below a given threshold (E-value≤1e–1). All selected sequences were subsequently ranked by *e*-value in ascending order, reserving the top S (default S = 10) of these sorted sequences, which contained rich evolution information. (If the number of selected sequences was less than S, all selected sequences were kept). The DHB elements in the matched unions were scored in three boxes that contained the profiles of three-state (DHB) information. The boxes constituted DHB profiles of the original sequence. The profile was clearly the frequency of the DHB elements, with three elements for each amino acid in the sequences. For the position *p* of an amino acid in the target, the normalized *s* profile (*s* = domain, hinge or boundary) was counted as
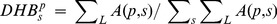
(1)where *p* was the position of the amino acid in the target sequence and *s* was one of the three DHB states. *A*(*p,s*) equaled 1 when the state of the *p* amino acid in the matched sequences was *s*. *L* was the number of matched sequences. In the denominator, the summation was carried out for three states.

The DHB of each amino acid had three elements: domain, hinge and boundary profiles. The DHB was a distinctive PSSM-like profile composed after alignment, with rich DHB information. Finally, the DHB was used as a feature to identify boundary regions in sequences.

### DomHR Architecture

**Figure 2:**
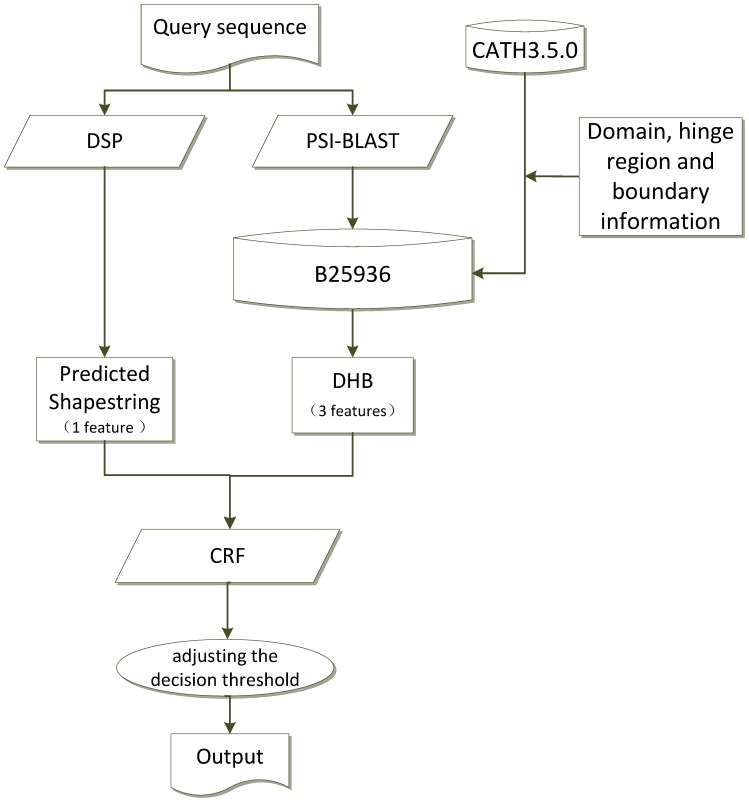
The flowchart of the DomHR. DSP (a protein shape string and its profile prediction server) and PSI-BLAST are carried out to generate predicted shape string features and DHR features. B25936 is constructed from CATH3.5.0 and domain, hinge region and boundary information.

The architecture of DomHR is shown in Figure 2. DomHR used two kinds of input: DHB profiles and predicted shape strings, for four total features. Three digital type features represented the DHB profile, which were obtained by BLAST alignment against B25936 and containing the homologous information of domain, hinge and boundary regions. One character type feature represented the predicted shape string, which is a sequence of particular shape symbols, one per residue, represented by a one-dimensional structural alphabet The shape string was initially proposed by Ison et al. [43] by clustering Φ*/Ψ* torsion angle pairs of protein backbones in a Ramachandran space into eight distinct regions, and assigning these clusters as eight symbols to describe the protein backbone. The real-value of protein backbone torsion angle has been predicted by Real-SPINE [44]. Shape string is a useful feature that has been applied by many studies such as for β-turn [45] and γ-turn prediction [46]. The shape string of a target protein sequence in this study was predicted by DSP (a protein shape string and its profile prediction server) [47].

The CRFs (Conditional Random Fields) was used for modeling and prediction. CRFs offer several advantages over other machine learning methods, including the ability to relax strong independence assumptions made in those models and avoid the fundamental limitation of maximum entropy Markov models and other discriminative Markov models based on directed graphical models; these can be biased towards states with few successor states [48] (See Text S1). CRFs also solve the label bias problem in a principled way, and they are faster than many other machine learning methods. In our approach, CRFs were used to model and predict without a slide window of sequences. We used a Unigram template that considered four upward variables and four downward variables in a row, and then all column variables were traversed. All modeling parameters were set to default. We used the CRF++ binary package for MS-Windows (CRF++0.54 is available at: http://crfpp.sourceforge.net/). The environment for training and testing was a Windows 7 64-bit operating system with Intel Core 2 Quad CPU and RAM 6 GB.

### Measuring Performance

To evaluate predictor performance, we used multiple measurements that are seldom used in the literature for predicting protein domain boundaries, for a comprehensive assessment. We used sensitivity [Sn = TP/(TP+FN)], specificity [Sp = TN/(TN+FP)], accuracy [Ac = (TP+TN)/(TP+FN+TN+FP)], Matthews’s correlation coefficient (MCC), weighted score (Sw), and area under the receiver operating characteristic (ROC) curve (AUC)
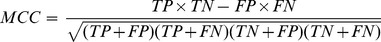
(2)

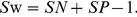
(3)where TP, FP, TN, and FN denote true positives, false positives, true negatives and false negatives; Sw and MCC ranged between -1 and 1 where 1 represented a perfect prediction and -1 represented a completely incorrect prediction. The statistical significance of the evaluation scores was determined by bootstrapping: 80% of the targets were randomly selected 1000 times, and the standard error of the scores was calculated.

## Results and Discussion

### Determining the Hinge Region Size

In this study, S628 was used to demonstrate the novel DHB feature that was generated using a BLAST tool modified from PSI-BLAST by aligning a query against B25936. Because B25936 was derived from a large and diverse database, when the BLAST function was performed, any sequence in B25936 with an exact match in S628 was discarded for fairness. To determine applicable size of the hinge region we assessed prediction performances dependent on hinge region size (Table 1) (See Table S1, S2). Residues in the predicted hinge region were determined to be classified into domain or boundary according to a decision threshold (see below). When R was set to 10, the Sw achieved the maximum, Sn was balanced with Sp, and AUC is also good. In our study, the size of the hinge region was always set as 10 for balance.

**Table 1:** Effects of size of hinge region on performance.

R value	Sn	Sp	MCC	Ac	Sw	AUC
**8**	0.78	0.90	0.46	0.90	0.67	0.90
**10**	0.81	0.87	0.43	0.86	0.68	0.89
**15**	0.84	0.80	0.37	0.81	0.65	0.88

### Validation of Features

A good feature greatly improves the accuracy of a predictor. To validate and select an optimal combination of features, 10 fold cross-validation tests were performed on S628. We randomly divided the S628 dataset into 10 subsets on average. One subset was used as a testing set, while the other nine subsets were merged into a training set. Table 2 shows the effects of different combination of features (See Table S3, S4). Since only DHB led to 0.69 for Sn, 0.88 for Sp, and 0.86 for Ac, 0.88 for AUC, DHB appeared to be an excellent feature when compared with reported features. We tested combinations of features such as predicted shape string (SST), predicted second structure (ss, 3-class secondary structure predicted by SPSSMPred [49]), profile of shape string (SSTP), profile of second structure (ssp), and PSSM. These often appear in the literature and were confirmed improve the prediction of protein structure and function. The DHB feature was combined with each and the effect was assessed. Table 2 shows that the SST was an effective companion feature for DHB, increasing Sn by 13% while Sp was reduced by 1% and all other measurement values increased. Although the results were same good when all features were used, too many features require additional computational time. Therefore, we selected only two features (DHB+SST) for our predictor.

**Table 2:** Performance comparison with different features on S628.

Combination of features	Sn	Sp	MCC	Ac	Sw	AUC
**DHB**	0.69	0.88	0.38	0.86	0.57	0.88
**DHB+SST**	0.82	0.87	0.45	0.87	0.69	0.89
**DHB+ss**	0.69	0.88	0.37	0.86	0.56	0.88
**DHB+SSTP**	0.81	0.87	0.44	0.87	0.68	0.88
**DHB+ssp**	0.69	0.88	0.38	0.86	0.57	0.88
**DHB+PSSM**	0.71	0.87	0.38	0.86	0.59	0.89
**DHB+SST+SSTP+** **ss+ssp+PSSM**	0.82	0.86	0.43	0.86	0.68	0.89

### Impact on Performance by Adjusting the Decision Threshold

S628 was used to demonstrate the hinge region strategy. The definition of the new hinge region improved the imbalance problem, and the ratio of the number of boundary residues to the number of domain residues changed from 1∶15 to 0.14∶1:4.80 for boundary:hinge:domain. Thus, a two-class problem was transformed to a three-class problem.

**Figure 3:**
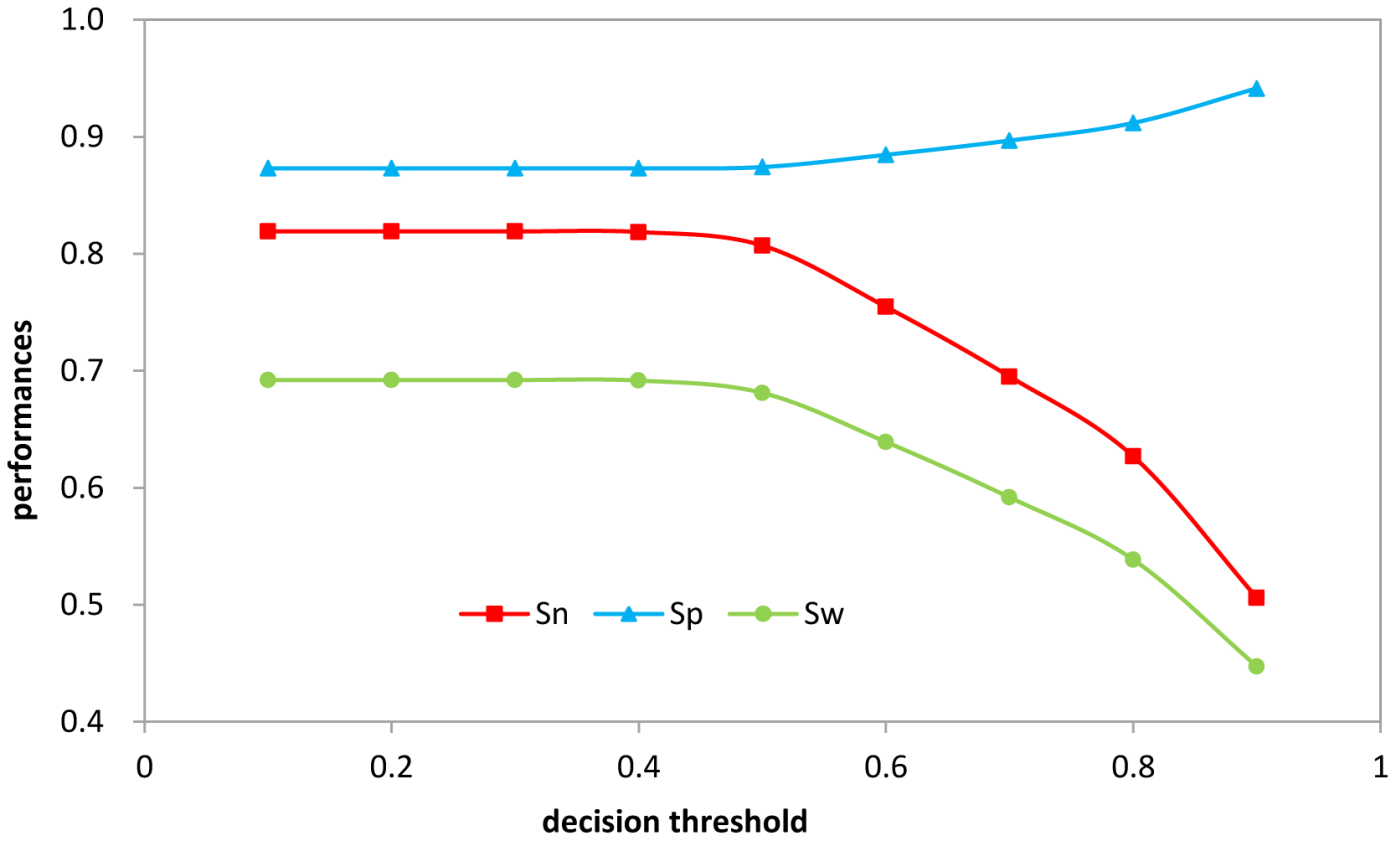
The performances in S628 by adjusting the different threshold values. The residues in the predicted hinge regions are determined to be either domain or boundary according to a decision threshold. If the probability on hinge regions of the residue was greater than a given threshold, the residue would be determined as boundary; otherwise it would belong to the domain.

Second, because the hinge region was defined to overlap parts of the domain and boundary regions, we determined the residues in the predicted hinge regions to be either domain or boundary according to a decision threshold that assigned a residue to the boundary or to the domain. The distributions of the probabilities at each residue position in the hinge region are provided in Figure S1. The results of adjusting decision thresholds are in Figure 3. When the decision threshold was 0.4, we achieved a maximum Sw, which was used as the criterion for optimization of the decision threshold.

**Figure 4:**
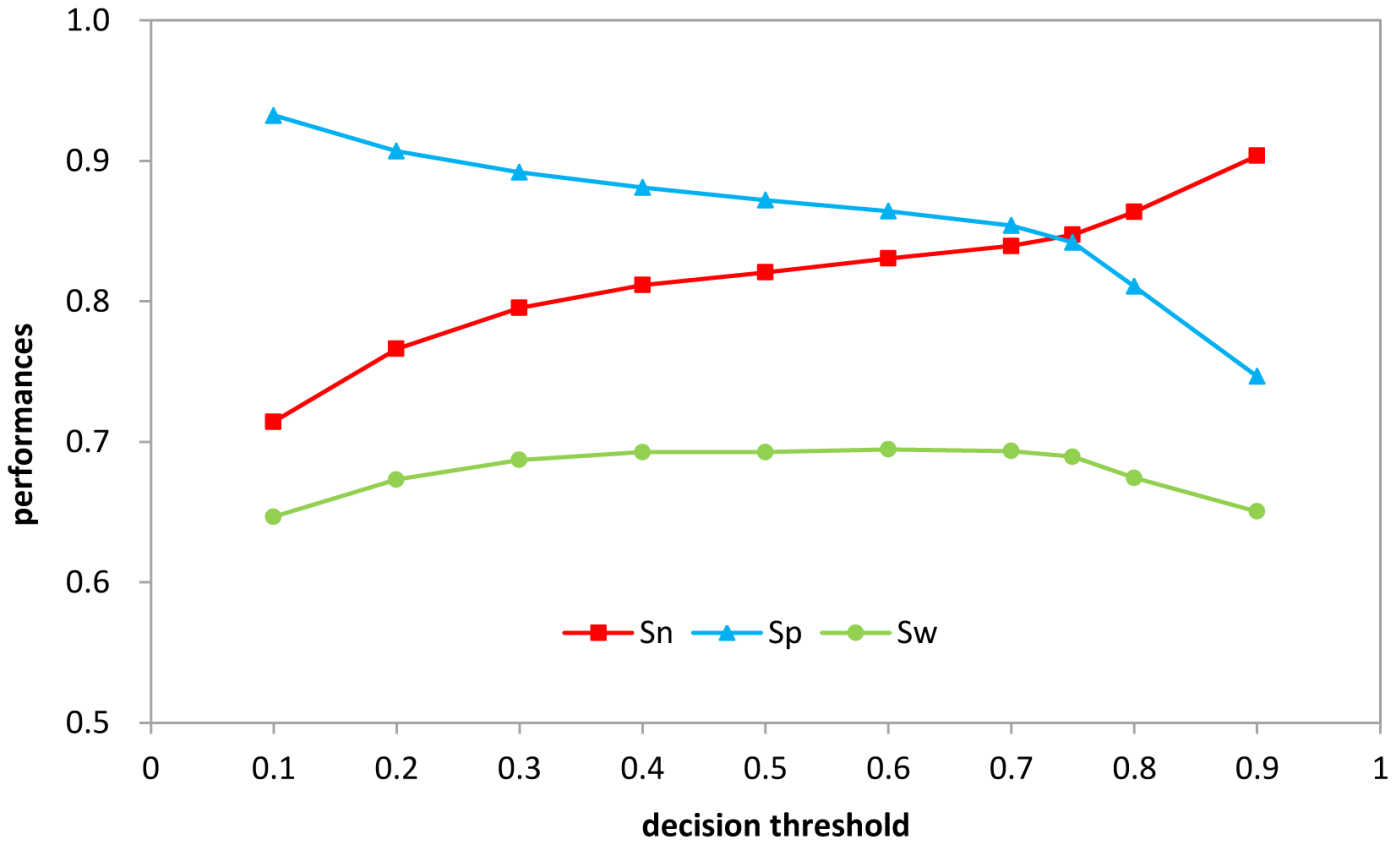
The performances in S628 by only adjusting threshold values of negative set. If the probability on domain regions of a residue was greater than a given threshold, the residue would be determined as domain; otherwise it would belong to the boundary.

Third, another feasible adjustment of decision threshold focused on the predicted domain regions. The decision threshold of the predicted domain regions could be adjusted to optimize sensitivity and specificity (Figure 4). If a probability of a residue was greater than a given threshold, the residue would be determined as domain; otherwise it would belong to the boundary. The Sw was at maximum when the decision threshold was 0.75.

Finally, we combined the adjustments in the predicted hinge and domain regions. The results showed no significant improvement, so the adjustments of the decision thresholds in the hinge and domain regions were both effective and could be used flexibly.

According to our results, adjusting the hinge probability did not substantially affect Sw greatly, however this worked well in blind testing. The adjustment of the domain probability greatly improved Sn and Sp. When the test was similar to the training, Sw achieved or approximated the maximum. However, when the blind testing was very different from the training, the optimized decision threshold based on the training did not guarantee the maximum Sw.

To summarize, the hinge region strategy proposed in this study had three central features: (i) definition; (ii) DHB feature; and (iii) adjusting the decision threshold. The hinge region strategy is an innovative technology that was confirmed to improve protein domain boundary identification.

### Validations of Large-scale Predictions

To understand the performance of the DomHR on large-scale prediction, we carried out a 10 fold cross-validation on S3845, an ensemble nonredundant set of CATH 3.4.0. The features used were DHB and predicted shape string, with a decision threshold of 0.4 in the predicted hinge region optimized based on the S628. S3845 was used as training set and S1508 as the independent testing set. Any sequence in B25936 with an exact match in S1508 was discarded for fairness. The results are in Table 3 (See Table S5, S6). The accurate performances confirmed that our predictor was outstanding even with large-scale validation. For S3845, we achieved an Sn of 0.80 and an Sp of 0.88. For S1508, we achieved an Sn of 0.77 and an Sp of 0.89. Table 3 also shows the results of one-domain, two-domain and multi-domain sequences contained in S1508. We achieved an Sn of 0.80 and an Sp of 0.91 for one-domain sequences, an Sn of 0.77 and an Sp of 0.89 for two-domain sequences, and an Sn of 0.71 and an Sp of 0.86 for multidomain sequences. These demonstrated that our approach had a strong ability to predict multidomain boundaries. All predicted results of S1508 are listed in Table S7.

**Table 3:** Performance on large-scale prediction.

Test dataset	Sn	Sp	MCC	Ac	Sw	AUC
**S3845^a^**	0.80	0.88	0.45	0.88	0.69	0.91
**S1508^b^**	0.77	0.89	0.45	0.88	0.66	0.91
**1-domain^c^**	0.80	0.91	0.51	0.90	0.70	0.92
**2-domain^c^**	0.77	0.89	0.46	0.89	0.66	0.91
**m-domain^c^**	0.71	0.86	0.35	0.85	0.57	0.88

a: ten-fold cross-validation of 3845 entries.

b: independent test (1508 entries) by training on the entire S3845.

c: sequences inS1508.

Two difficulties hindered comparison of our approach with previous studies. One is the small number of comprehensive assessments of published approaches. The other is that the datasets used were different, and lacked large-scale results. We compared DomHR with DoBo [5], and based on S628 and 10 fold cross-validation, our Sn of 0.82 was 8% higher than that of DoBo at 0.74. Because DoBo stated that “a predicted domain boundary is regarded as correct when the predicted boundary fully or partially overlaps the true (official) boundary within a margin of tolerance of ±20 residues,” the Sp of DoBo should be low.

In addition, we compared DomHR with DomSSEA [24] which was a fast alignment-based domain prediction method. The reseacher found that DomSSEA correctly identified the number of domains in 72% of all proteins tested, and correctly identified 24% of all domain boundaries within ±20 residues of the boundaries annotated in CATH [28]. So our Ac of 87% was far higher than DomSSEA.

For large-scale prediction, we compared DomHR with Cai [37] for about 1508 entries vs. 1299 entries. Our Sn of 0.77 was 13% higher than Cai’s of 0.64, and our Sp of 0.89 was 8% higher than Cai’s 0.81. Other evaluation values of our approach were also better than Cai’s.

Improving the accuracy in predicting multidomain boundaries is a challenging task because the accuracy usually is considerably less than 40% [35]. We assessed the performances of DomHR on single-domain, two-domain, and multidomain sequences in the S1508 set (Table 3), and the results showed that our approach was a balanced predictor with an excellent ability to predict single-domain, two-domain and multidomain boundaries. The Sn of 0.71 indicated unprecedented accuracy in multidomain boundary prediction.

### Prediction of CASP 9

CASP 9 is a challenging subset for domain boundary prediction.

Zhou [42] had good results using an optimized decision threshold based on the CASP 8 set to predict regions of protein disorder. We carried out DomHR on a blind test of CASP 9, using CASP 8 set as training set. DHB feature and predicted shape string were used as features, and any sequence in B25936 with an exact match in CASP 9 was discarded for fairness. The domain region decision threshold was optimized based on the S628 (0.75). The results are in Table 4 (See Table S8, S9). The Sn was 0.73, Sp was 0.59 and Sw was 0.33. Comparison with published results indicated the accuracy of prediction of CASP 9. DoBo evaluated only 14 targets in CASP 9, with a result of an Sn of 0.70. Results were Sn of 0.52 in PPRODO and Sn of 0.14 in DOMPro. This demonstrated the effectiveness of our approach.

**Table 4:** Performance on CASP9 trained on CASP8.

Test dataset	Sn	Sp	MCC	Ac	Sw	AUC
**CASP9**	0.73	0.59	0.20	0.60	0.33	0.75
**1-domain^d^**	0.75	0.63	0.24	0.64	0.37	0.77
**m-domain^d^**	0.70	0.57	0.16	0.59	0.28	0.71

d: sequences in CASP 9.

Because most single domains in CASP 9 were discontinuous, as multidomain sequences, predicting single domains was as difficult as predicting multiple domains. The difference in prediction accuracy between single-domain and multidomain proteins was not large. For the multidomain CASP 9, the Sn from DoBo was 0.68, while our Sn was 0.70.

In this study, CASP 9 was considered to be a blind test, so we did not adjust the decision threshold according the performance of the CASP 9 prediction to achieve a reasonable Sn and Sp. Because the decision threshold optimization was based on S628, the specificities in Table 4 were lower than the sensitivities. This indicated more research needs to be done in this field. All predicted results of CASP 9 are available in Table S10.

### Web Servers

We have created a DomHR server for scientific users on our local infrastructure. This is available at http://cal.tongji.edu.cn/domain/. The DomHR server predicts protein domain boundary for query sequence(s), and results are provided in the form of a web page and/or an e-mail.

### Conclusion

In this work, we proposed a hybrid method, DomHR, to accurately predict domain boundaries in proteins based on a creative hinge region strategy. DomHR was tested in cross-validation, large-scale prediction, independent tests, and CASP tests. All results confirmed that DomHR outperformed well-established publicly available domain boundary predictors for accuracy of prediction. The kernel technology is a hinge region strategy that generates an effective feature: a DHB feature by alignment. For the idea, it came from the fact that the most predicted errors existed in the connecting regions of domains and boundaries. We considered firstly the new defined hinge region would reduce the imbalance of dataset. Then, the proposed DHB feature can accurately predict the hinge regions. At the last, the adjustments of the decision thresholds of the predicted hinge regions were local adjustments, which could help to optimize the performance of the predictor. However, similar to other alignment-based methods, the performance of DomHR requires homology information obtained when the alignment is carried out. The most distinct characteristic of DomHR was that only four features were used during modeling and prediction. This number is much lower than previous methods. A few features and high accuracy make our approach competitive with state-of-the-art domain boundary predictors. We propose that as more structures of proteins are determined, obtaining homology information will become easier. We also believe this strategy benefits other predictions of protein structure and function.

## Supporting Information

Figure S1 **The distributions of the probabilities at each residue position in the hinge region.** The negative values on the abscissa refer to residue positions in the hinge region close to domain region, and the positive values on the abscissa refer to residue positions in the hinge region close to boundary region. The values on the ordinate refer to average probability at each residue position in the hinge region. The probabilities at residue positions close to boundary region are generally higher than those close to domain region.
(TIF)

Table S1 **Effects of size of hinge region on performance (TP, FN, TN and FP).**
(DOCX)

Table S2 **Effects of size of hinge region on performance (including SE).**
(DOCX)

Table S3 **Performance comparison with different features on S628 (TP, FN, TN and FP).**
(DOCX)

Table S4 **Performance comparison with different features on S628 (including SE).**
(DOCX)

Table S5 **Performance on large-scale prediction (TP, FN, TN and FP).**
(DOCX)

Table S6 **Performance on large-scale prediction (including SE).**
(DOCX)

Table S7 **All predicted results of S1508.**
(XLSX)

Table S8 **Performance on CASP9 trained on CASP8 (TP, FN, TN and FP).**
(DOCX)

Table S9 **Performance on CASP9 trained on CASP8 (including SE).**
(DOCX)

Table S10 **All predicted results of CASP 9.**
(XLSX)

Text S1 **Detailed descriptions of CRF (Conditional Random Fields) methodology.**
(DOCX)
