# Supplementary figures and images for: DomHR: Accurately Identifying Domain Boundaries in Proteins Using a Hinge Region Strategy
Source: PLoS One. 2013 Apr 11;8(4):e60559. doi: 10.1371/journal.pone.0060559 (PMC3623903; doi:10.1371/journal.pone.0060559)

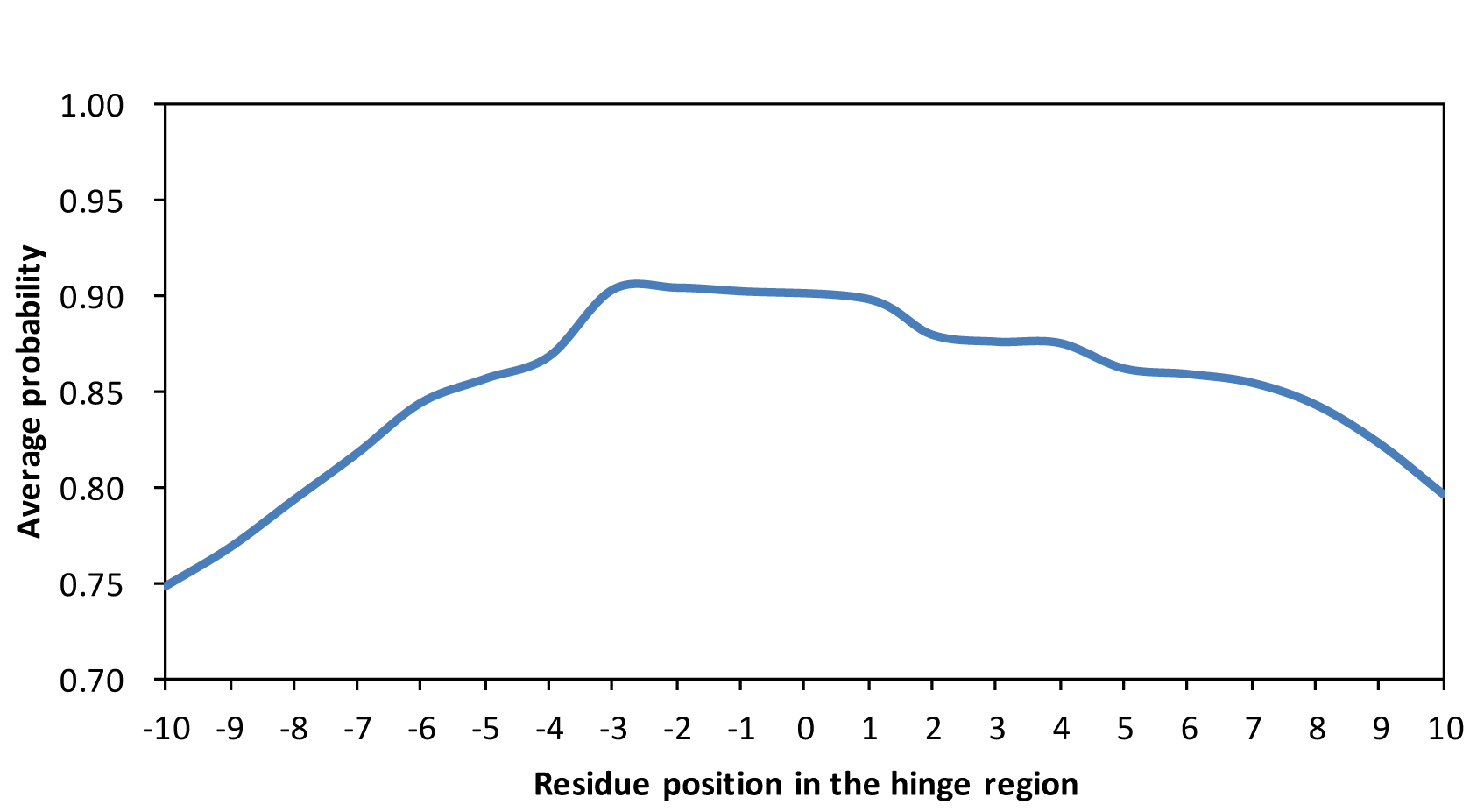

Supplement: Figure S1 — The distributions of the probabilities at each residue position in the hinge region. The negative values on the abscissa refer to residue positions in the hinge region close to domain region, and the positive values on the abscissa refer to residue positions in the hinge region close to boundary region. The values on the ordinate refer to average probability at each residue position in the hinge region. The probabilities at residue positions close to boundary region are generally higher than those close to domain region. (TIF) [file pone.0060559.s001.tif]
