# Supplementary material for: DomHR: Accurately Identifying Domain Boundaries in Proteins Using a Hinge Region Strategy
Source: PLoS One. 2013 Apr 11;8(4):e60559. doi: 10.1371/journal.pone.0060559 (PMC3623903; doi:10.1371/journal.pone.0060559)
Supplement: Table S1 — Effects of size of hinge region on performance (TP, FN, TN and FP). (DOCX) [file pone.0060559.s002.docx]

Supporting Information Table S1

Table S1: Effects of size of hinge region on performance (TP, FN, TN and FP)

| R value | TP | FN | TN | FP |
| --- | --- | --- | --- | --- |
| 8 | 6446 | 1837 | 111306 | 12800 |
| 10 | 6682 | 1601 | 107573 | 16533 |
| 15 | 6983 | 1300 | 99889 | 24217 |
