# Supplementary material for: DomHR: Accurately Identifying Domain Boundaries in Proteins Using a Hinge Region Strategy
Source: PLoS One. 2013 Apr 11;8(4):e60559. doi: 10.1371/journal.pone.0060559 (PMC3623903; doi:10.1371/journal.pone.0060559)
Supplement: Table S2 — Effects of size of hinge region on performance (including SE). (DOCX) [file pone.0060559.s003.docx]

Supporting Information Table S2

Table S2: Effects of size of hinge region on performance (including SE)

|  | Sn | |  | Sp | |  | MCC | |  | Ac | |  | Sw | |  |  |
| --- | --- | --- | --- | --- | --- | --- | --- | --- | --- | --- | --- | --- | --- | --- | --- | --- |
| R | value | ±SE |  | value | ±SE |  | value | ±SE |  | value | ±SE |  | value | ±SE |  | AUC |
| 8 | 0.7772 | 0.0209 |  | 0.8970 | 0.0013 |  | 0.4638 | 0.0100 |  | 0.8895 | 0.0018 |  | 0.6742 | 0.0208 |  | 0.8970 |
| 10 | 0.8115 | 0.0171 |  | 0.8664 | 0.0014 |  | 0.4304 | 0.0079 |  | 0.8630 | 0.0017 |  | 0.6779 | 0.0171 |  | 0.8926 |
| 15 | 0.8412 | 0.0160 |  | 0.8044 | 0.0022 |  | 0.3688 | 0.0058 |  | 0.8067 | 0.0023 |  | 0.6457 | 0.0162 |  | 0.8780 |
