# Supplementary material for: DomHR: Accurately Identifying Domain Boundaries in Proteins Using a Hinge Region Strategy
Source: PLoS One. 2013 Apr 11;8(4):e60559. doi: 10.1371/journal.pone.0060559 (PMC3623903; doi:10.1371/journal.pone.0060559)
Supplement: Table S3 — Performance comparison with different features on S628 (TP, FN, TN and FP). (DOCX) [file pone.0060559.s004.docx]

Supporting Information Table S3

Table S3: Performance comparison with different features on S628 (TP, FN, TN and FP)

| Combination of features | TP | FN | TN | FP |
| --- | --- | --- | --- | --- |
| DHB | 5741 | 2542 | 108706 | 15400 |
| DHB+SST | 6784 | 1499 | 108345 | 15761 |
| DHB+ss | 5737 | 2546 | 108732 | 15374 |
| DHB+SSTP | 6680 | 1603 | 108546 | 15560 |
| DHB+ssp | 5720 | 2563 | 108666 | 15440 |
| DHB+PSSM | 5909 | 2374 | 108162 | 15944 |
| DHB+SST+SSTP+ss+ssp+PSSM | 6776 | 1507 | 107163 | 16943 |
