# Supplementary material for: DomHR: Accurately Identifying Domain Boundaries in Proteins Using a Hinge Region Strategy
Source: PLoS One. 2013 Apr 11;8(4):e60559. doi: 10.1371/journal.pone.0060559 (PMC3623903; doi:10.1371/journal.pone.0060559)
Supplement: Table S4 — Performance comparison with different features on S628 (including SE). (DOCX) [file pone.0060559.s005.docx]

Supporting Information Table S4

Table S4: Performance comparison with different features on S628 (including SE)

| Combination | Sn | |  | Sp | | |  | | MCC | | |  | | Ac | | |  | | Sw | | |  | |  | |  |
| --- | --- | --- | --- | --- | --- | --- | --- | --- | --- | --- | --- | --- | --- | --- | --- | --- | --- | --- | --- | --- | --- | --- | --- | --- | --- | --- |
| of features | value | ±SE | |  | value | ±SE | |  | | value | ±SE | |  | | value | ±SE | |  | | value | ±SE | |  | | AUC | |
| DHB | 0.6975 | 0.0193 | |  | 0.8756 | 0.0014 | |  | | 0.3777 | 0.0075 | |  | | 0.8644 | 0.0018 | |  | | 0.5730 | 0.0190 | |  | | 0.8793 | |
| DHB+SST | 0.8214 | 0.0173 | |  | 0.8731 | 0.0013 | |  | | 0.4472 | 0.0074 | |  | | 0.8697 | 0.0017 | |  | | 0.6946 | 0.0174 | |  | | 0.8853 | |
| DHB+ss | 0.6884 | 0.0143 | |  | 0.8763 | 0.0014 | |  | | 0.3743 | 0.0053 | |  | | 0.8644 | 0.0017 | |  | | 0.5647 | 0.0141 | |  | | 0.8770 | |
| DHB+SSTP | 0.8087 | 0.0166 | |  | 0.8744 | 0.0013 | |  | | 0.4412 | 0.0075 | |  | | 0.8703 | 0.0015 | |  | | 0.6831 | 0.0164 | |  | | 0.8795 | |
| DHB+ssp | 0.6909 | 0.0145 | |  | 0.8758 | 0.0015 | |  | | 0.3751 | 0.0064 | |  | | 0.8641 | 0.0020 | |  | | 0.5667 | 0.0148 | |  | | 0.8764 | |
| DHB+PSSM | 0.7151 | 0.0184 | |  | 0.8716 | 0.0012 | |  | | 0.3818 | 0.0073 | |  | | 0.8618 | 0.0017 | |  | | 0.5867 | 0.0184 | |  | | 0.8873 | |
| DHB+SST+SSTP+ss+ssp+PSSM | 0.8208 | 0.0190 | |  | 0.8634 | 0.0014 | |  | | 0.4314 | 0.0086 | |  | | 0.8607 | 0.0017 | |  | | 0.6842 | 0.0189 | |  | | 0.8899 | |
