# Supplementary material for: DomHR: Accurately Identifying Domain Boundaries in Proteins Using a Hinge Region Strategy
Source: PLoS One. 2013 Apr 11;8(4):e60559. doi: 10.1371/journal.pone.0060559 (PMC3623903; doi:10.1371/journal.pone.0060559)
Supplement: Table S5 — Performance on large-scale prediction (TP, FN, TN and FP). (DOCX) [file pone.0060559.s006.docx]

Supporting Information Table S5

Table S5: Performance on large-scale prediction (TP, FN, TN and FP)

| Test | TP | FN | TN | FP |
| --- | --- | --- | --- | --- |
| S3845^a^ | 45187 | 11005 | 761482 | 100454 |
| S1510^b^ | 17741 | 5288 | 299267 | 37028 |
| 1-domain^c^ | 10530 | 2683 | 164692 | 16868 |
| 2-domain^c^ | 2834 | 846 | 48756 | 5805 |
| m-domain^c^ | 4375 | 1761 | 85819 | 14355 |

a: ten-fold cross-validation of 3845 entries.

b: independent test (1510 entries) by training on the entire S3845.

c: sequences inS1510.
