# Supplementary material for: DomHR: Accurately Identifying Domain Boundaries in Proteins Using a Hinge Region Strategy
Source: PLoS One. 2013 Apr 11;8(4):e60559. doi: 10.1371/journal.pone.0060559 (PMC3623903; doi:10.1371/journal.pone.0060559)
Supplement: Table S6 — Performance on large-scale prediction (including SE). (DOCX) [file pone.0060559.s007.docx]

Supporting Information Table S6

Table S6: Performance on large-scale prediction (including SE)

|  | Sn | |  | Sp | |  | MCC | |  | Ac | |  | Sw | |  |  |
| --- | --- | --- | --- | --- | --- | --- | --- | --- | --- | --- | --- | --- | --- | --- | --- | --- |
| Test | value | ±SE |  | value | ±SE |  | value | ±SE |  | value | ±SE |  | value | ±SE |  | AUC |
| S3845^a^ | 0.8042 | 0.0032 |  | 0.8834 | 0.0007 |  | 0.4511 | 0.0021 |  | 0.8786 | 0.0007 |  | 0.6876 | 0.0032 |  | 0.9148 |
| S1508^b^ | 0.7710 | 0.0067 |  | 0.8899 | 0.0011 |  | 0.4503 | 0.0036 |  | 0.8823 | 0.0011 |  | 0.6609 | 0.0064 |  | 0.9086 |
| 1-domain^c^ | 0.7969 | 0.0075 |  | 0.9071 | 0.0011 |  | 0.5094 | 0.0042 |  | 0.8996 | 0.0011 |  | 0.7040 | 0.0074 |  | 0.9201 |
| 2-domain^c^ | 0.7690 | 0.0084 |  | 0.8942 | 0.0017 |  | 0.4585 | 0.0044 |  | 0.8861 | 0.0020 |  | 0.6633 | 0.0092 |  | 0.9140 |
| m-domain^c^ | 0.7126 | 0.0133 |  | 0.8573 | 0.0025 |  | 0.3495 | 0.0053 |  | 0.8489 | 0.0024 |  | 0.5699 | 0.0128 |  | 0.8801 |

a: ten-fold cross-validation of 3845 entries.

b: independent test (1508 entries) by training on the entire S3845.

c: sequences inS1508.
