# Supplementary material for: DomHR: Accurately Identifying Domain Boundaries in Proteins Using a Hinge Region Strategy
Source: PLoS One. 2013 Apr 11;8(4):e60559. doi: 10.1371/journal.pone.0060559 (PMC3623903; doi:10.1371/journal.pone.0060559)
Supplement: Table S8 — Performance on CASP9 trained on CASP8 (TP, FN, TN and FP). (DOCX) [file pone.0060559.s009.docx]

Supporting Information Table S8

Table S8: Performance on CASP9 trained on CASP8 (TP, FN, TN and FP)

| Test | TP | FN | TN | FP |
| --- | --- | --- | --- | --- |
| CASP9 | 2092 | 747 | 13663 | 9512 |
| 1-domain^d^ | 1573 | 522 | 9806 | 6047 |
| m-domain^d^ | 519 | 225 | 3857 | 3465 |

d: sequences in CASP 9.
