# Supplementary material for: DomHR: Accurately Identifying Domain Boundaries in Proteins Using a Hinge Region Strategy
Source: PLoS One. 2013 Apr 11;8(4):e60559. doi: 10.1371/journal.pone.0060559 (PMC3623903; doi:10.1371/journal.pone.0060559)
Supplement: Table S9 — Performance on CASP9 trained on CASP8 (including SE). (DOCX) [file pone.0060559.s010.docx]

Supporting Information Table S9

Table S9: Performance on CASP9 trained on CASP8 (including SE)

|  | Sn | |  | Sp | |  | MCC | |  | Ac | |  | Sw | |  |  |
| --- | --- | --- | --- | --- | --- | --- | --- | --- | --- | --- | --- | --- | --- | --- | --- | --- |
| Test | value | ±SE |  | value | ±SE |  | value | ±SE |  | value | ±SE |  | value | ±SE |  | AUC |
| CASP9 | 0.7340 | 0.0124 |  | 0.5871 | 0.0189 |  | 0.2042 | 0.0111 |  | 0.6037 | 0.0161 |  | 0.3271 | 0.0201 |  | 0.7519 |
| 1-domain^d^ | 0.7473 | 0.0112 |  | 0.6273 | 0.0071 |  | 0.2374 | 0.0067 |  | 0.6405 | 0.0053 |  | 0.3746 | 0.0093 |  | 0.7672 |
| m-domain^d^ | 0.7022 | 0.0086 |  | 0.5732 | 0.0230 |  | 0.1640 | 0.0129 |  | 0.5855 | 0.0208 |  | 0.2754 | 0.0144 |  | 0.7051 |

d: sequences in CASP 9.
