# Supplementary material for: DomHR: Accurately Identifying Domain Boundaries in Proteins Using a Hinge Region Strategy
Source: PLoS One. 2013 Apr 11;8(4):e60559. doi: 10.1371/journal.pone.0060559 (PMC3623903; doi:10.1371/journal.pone.0060559)
Supplement: Text S1 — Detailed descriptions of CRF (Conditional Random Fields) methodology. (DOCX) [file pone.0060559.s012.docx]

Supporting Information Text S1

**Detailed descriptions of CRF (Conditional Random Fields) methodology**

**Brief description of Conditional Random Fields**

The machine learning method we used is Conditional Random Fields (CRFs) and the software of CRF is CRF++0.54 which is available at: <http://crfpp.sourceforge.net/>. The principle of CRF is described in references [1,2], and has been used in bioinformatics recently. David [3] used CRF to predict gene. Wang [4] studied RNA sequence-structure relationship based on CRF method. CRF also were used to predict protein secondary structure [5,6].

**The format of CRF input file**

The coding mode is provided as following, which will be inputed into the CRF++ software. The first column are sequences expressed by amino acids. The second column are predicted shapestring features expressed by one character (8-state). The next three columns are DHB features expressed by DHB profile. The finally column is class labels (0 for domain, 1 for boundary and 2 for hinge region).


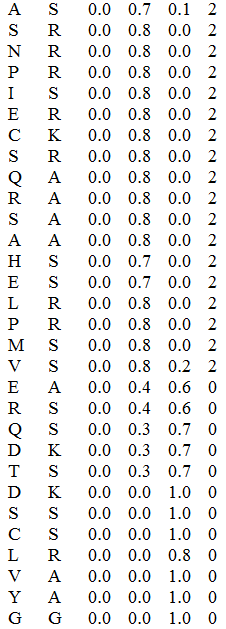


**The template file**

The template is provided as following, which will be used to training in the CRF++ software.


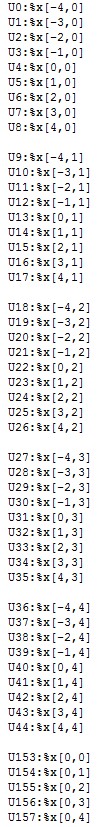


**References:**

[1] Lafferty, J.D. et al. (2001) Conditional random fields: probabilistic models for segmenting and labeling sequence data. In ICML 2001: Proceedings of the Eighteenth International Conference on Machine Learning; 28 June–1 July, 2001. Morgan Kaufmann Publishers Inc., San Francisco, CA, USA, pp. 282–289

[2] Sutton, C. and McCallum, A. (2006) An introduction to conditional random fields for relational learning. In Introduction to statistical relational learning (eds. L. Getoor and B. Taskar), MIT Press, Cambridge, MA, pp. 1–35

[3] David DeCaprio, Jade P. Vinson, Matthew D. Pearson, et al.(2008) Conrad: Gene prediction using conditional random fields, Genome Research, 17: 1389–1398

[4] Zhiyong Wang and Jinbo Xu. A conditional random fields method for RNA sequence–structure relationship modeling and conformation sampling, Bioinformatics,Vol. 27 ISMB 2011, pages i102–i110

[5] Li,D., Li,T., Cong,P., Xiong,W. and Sun,J. (2012) A novel structural position-specific scoring matrix for the prediction of protein secondary structures. Bioinformatics, 28: 32–39

[6] Zhiyong Wang, Feng Zhao, Jian Peng and Jinbo Xu.(2011) Protein 8-class secondary structure prediction using conditional neural fields, Proteomics, 11: 3786–3792
